# A MENDELIAN RANDOMIZATION STUDY SUPPORTS CAUSAL EFFECT OF OVERWEIGHT ON LONGEVITY

**DOI:** 10.1093/geroni/igad104.2909

**Published:** 2023-12-21

**Authors:** Hongzhe Duan, Rachel Holmes, Konstantin Arbeev, Olivia Bagley, Deqing Wu, Anatoliy Yashin, Svetlana Ukraintseva

**Affiliations:** Social Science Research Institute, Duke University, Durham, North Carolina, United States; Social Science Research Institute, Duke University, Durham, North Carolina, United States; Social Science Research Institute, Duke University, Durham, North Carolina, United States; Social Science Research Institute, Duke University, Durham, North Carolina, United States; Social Science Research Institute, Duke University, Durham, North Carolina, United States; Social Science Research Institute, Duke University, Durham, North Carolina, United States; Duke University, Durham, North Carolina, United States

## Abstract

Overweight was associated with better survival in some studies. This was termed “obesity paradox”; however, its mechanism is unclear. Mendelian Randomization (MR) is a common approach to uncovering causality in observational data using SNPs as instrumental variables (IVar). However, only few SNPs usually pass all the required assumptions. Here we use a new strategy of selecting IVar for the MR study of causal relationships between overweight and longevity in the Health and Retirement Study (HRS) data. We paired all SNPs in eight candidate obesity-related genes (ADIPOQ, FTO, LEP, LEPR, INSIG2, MC4R, PCSK1, PPARG) available in HRS, and used such ‘composite SNPs’ as IVar, which resulted in significantly more IVar candidates compared to single SNPs. The following variables were used in MR analysis as “exposure”: (1) ‘overweight’ BMI 25-30 vs. (0) ‘normal’ BMI 18.5-25, at ages 75-85; and “outcome”: (1) survived 85+ vs. (0) died before age 85. Inverse-Variance Weighted (IVW), and maximum likelihood (ML) from R-package MendelianRandomization were used to test causality. We found that being overweight at ages 75-85 had significant protective effect on survival at ages 85+ in both sexes, white males, and black males and females. In conclusion, pairing SNPs is a convenient simple approach to increasing numbers of IVar candidates for MR. Results of our MR study suggest that being overweight in late life causally contributes to longevity. One potential mechanism may involve larger energy reserves contributing to a better resilience of overweight people in face of an adverse health event, such as, e.g., major surgery.

